# Testing beat perception without sensory cues to the beat: the Beat-Drop Alignment Test (BDAT)

**DOI:** 10.3758/s13414-022-02592-2

**Published:** 2022-10-19

**Authors:** Urte Cinelyte, Jonathan Cannon, Aniruddh D. Patel, Daniel Müllensiefen

**Affiliations:** 1 Department of Psychology, Goldsmiths, University of London, London, UK; 2 Department of Psychology, Neuroscience and Behaviour, McMaster University, Hamilton, ON Canada; 3 Department of Psychology, Tufts University, Medford, MA USA; 4 Program in Brain, Mind, and Consciousness, Canadian Institute for Advanced Research (CIFAR), Toronto, ON Canada

**Keywords:** Music cognition, Beat perception, Sensorimotor abilities

## Abstract

Beat perception can serve as a window into internal time-keeping mechanisms, auditory–motor interactions, and aspects of cognition. One aspect of beat perception is the covert continuation of an internal pulse. Of the several popular tests of beat perception, none provide a satisfying test of this faculty of covert continuation. The current study proposes a new beat-perception test focused on covert pulse continuation: The Beat-Drop Alignment Test (BDAT). In this test, participants must identify the beat in musical excerpts and then judge whether a single probe falls on or off the beat. The probe occurs during a short break in the rhythmic components of the music when no rhythmic events are present, forcing participants to judge beat alignment relative to an internal pulse maintained in the absence of local acoustic timing cues. Here, we present two large (*N* > 100) tests of the BDAT. In the first, we explore the effect of test item parameters (e.g., probe displacement) on performance. In the second, we correlate scores on an adaptive version of the BDAT with the computerized adaptive Beat Alignment Test (CA-BAT) scores and indices of musical experience. Musical experience indices outperform CA-BAT score as a predictor of BDAT score, suggesting that the BDAT measures a distinct aspect of beat perception that is more experience-dependent and may draw on cognitive resources such as working memory and musical imagery differently than the BAT. The BDAT may prove useful in future behavioral and neural research on beat perception, and all stimuli and code are freely available for download.

**Supplementary Information:**

The online version contains sample audio files and supplementary material available at 10.3758/s13414-022-02592-2.

## Introduction

Musical beat perception involves inferring an underlying periodic pulse from an extract of music and anticipating the timing of each beat as the music unfolds (Patel & Iversen, 2014). Beat perception is a core aspect of music cognition and is a natural capacity in the general population (Phillips-Silver et al., 2011). However, significant variance is present in beat perception abilities (Sowiński & Dalla Bella, 2013). In some cases, individuals can demonstrate extreme difficulties performing tasks that require accurate beat tracking and synchronization (Palmer et al., 2014; Phillips-Silver et al., 2011), or show poor rhythm perception without any impairment in motor synchronization (Bégel et al., 2017).

Understanding the ability to perceive a musical beat can inform models of human time-keeping mechanisms and temporal adaptation (Palmer et al., 2014). These mechanisms have been linked to certain behavioural and cognitive traits (e.g., impulsivity; Allman & Meck, 2012) including attention levels and learning (Taatgen et al., 2007). Moreover, deficits in time perception are associated with a number of neurological and psychiatric conditions (e.g., Parkinson’s disease, schizophrenia, attention deficit hyperactivity disorder, and autism; Allman & Meck, 2012; Breska & Ivry, 2018; Grahn & Brett, 2009; Puyjarinet et al., 2017; Turgeon et al., 2012), and atypical rhythmic timing abilities are associated with some childhood language disorders (e.g., dyslexia, stuttering, developmental language disorder; Ladányi et al., 2020)

For these reasons, the evaluation of beat perception ability is of interest not only in education (Ladányi et al., 2020; Ozernov-Palchik et al., 2018) but also in clinical settings (Cochen De Cock et al., 2018), and beat-perception tests can serve as a useful tool in both contexts. The need for a reliable measure of beat perception is evident from the number of beat-perception tests developed over the past decade or so, including the Beat Alignment Test (BAT; Iversen & Patel, 2008), the Harvard Beat Assessment Test (H-BAT; Fujii & Schlaug, 2013), and the Battery for the Assessment of Auditory Sensorimotor Timing Abilities (BAASTA; Dalla Bella et al., 2017), which combines the BAT with several other perceptual and production-based measures of timing abilities. Notably, research with the BAASTA found that performance on the BAT was not correlated with tests of interval timing but was correlated with performance on paced tapping to a rhythmic stimulus, suggesting that the BAT is sensitive to beat-based timing mechanisms (Dalla Bella et al., 2017; cf. Fiveash et al., 2022). Recently, two updated versions of the BAT have been developed: the Adaptive Beat Alignment Test (A-BAT; Ross et al., 2018) and the Computerized Adaptive Beat Alignment Test (CA-BAT; Harrison & Müllensiefen, 2018). We note in passing that the rhythm and meter subtests of the Montreal Battery for Evaluation for Amusia (MBEA; Peretz et al., 2003) are not sensitive tests of beat perception abilities, as recently documented by Peretz and colleagues (Tranchant et al., 2021).

The human faculty of rhythm has recently been shown to be multidimensional: individuals’ rhythmic abilities vary along the independent axes of rhythm production, sequence-memory-based rhythm perception, and beat-based rhythm perception (Fiveash et al., 2022; Tierney & Kraus, 2015). But it is possible that even these subskills are composed of independent proficiencies. The process of identifying and maintaining a beat percept is not well understood, but it likely involves a complex interplay of processing auditory features, measuring durations, and internally producing similar durations, allowing the listener to continue to perceive a beat through complex rhythms in which not all beats are marked by sound events (Cannon & Patel, 2021). Existing beat-perception tests emphasize some of these aspects of beat perception at the expense of others. The BAT, for example, asks participants to determine whether a metronomic sequence of tones is on or off the beat of a musical excerpt. A participant could succeed by adopting a strategy of comparing the timing of the tones to the local acoustic features associated with the music’s beat; thus, recognizing the acoustic events associated with beats may be more important for passing this test than the ability to internally continue the pulse with accurate timing. Perceptual tasks using metronomic clicks or tones (e.g., the anisochrony detection tasks included in the BAASTA; Dalla Bella et al., 2017) emphasize the measurement and production of durations, but do not test the ability to continue a pulse drawn spontaneously from the complex acoustic features of music. This distinction may be quite important: non-isochrony detection could be performed by simple comparison of consecutive time intervals, and some participants may be able to synchronize with simple metronome clicks but lack the ability to extract a beat from music, as in the case of beat-deafness reported in Phillips-Silver et al. (2011).

This paper reports the development of the new Beat Drop Alignment Test (BDAT), which aims to bring the process of covert music-induced pulse continuation into focus. The BDAT employs naturalistic musical stimuli and asks participants to judge whether a single probe event falls on the musical beat or not. Crucially, the probe occurs within a bar of music from which all rhythmic cues have been removed after the first beat, eliminating the ability to do the task based on judging the alignment of the probe with locally prominent acoustic events. Hence, to perform well on the BDAT participants are required to (1) extract a mental representation of the musical beat, (2) continue this mental representation with accurate timing over a short period when there are no sensory cues to the beat, and (3) to compare their mental representation of the beat to a single probe sound.

In line with findings from the beat processing literature, we hypothesize that the difficulty of items on this test will mainly depend on three factors: the degree of probe displacement from the target beat (Harrison & Müllensiefen, 2018), the probe displacement direction in relation to the target beat (early/late; Van Der Steen et al., 2014), and the metrical strength of the target beat (Patel et al., 2005). In addition, we hypothesize that individuals with extensive training in music or dance will show superior performance on the BDAT task compared with the general public, and that performance on the BDAT will be associated with the level of musical experience (i.e., general musical sophistication; GMS) as measured by the Gold-MSI (Müllensiefen et al., 2014).

## Additional distinctive features of the BDAT

The BDAT has other appealing features from the standpoint of testing beat perception. First, it uses naturalistic music tracks. By using varied, complex instrumentation, the BDAT aims to maintain the listener’s interest and avoid the fatigue that can come from psychophysical timing tasks which use simple and timbrally uniform sounds on each trial. Second, it is quickly administered: The BDAT does not exceed 15 minutes and can be as short as 7 minutes. Because it was constructed as an adaptive test, testing length (number of items) and duration (in minutes) are flexible in order to ensure the highest testing efficiency in the shortest time. Third, the BDAT has unambiguously defined beat times. Since the BDAT stimuli were created specifically for the test (using a synthesizer), beat locations were based on the MIDI grid used to align the electronic instruments, and probe sounds were placed relative to these beat locations. Computer-generated stimuli have been previously used in BAASTA (Dalla Bella et al., 2017), but not in the BAT, A-BAT, or CA-BAT. Fourth, unlike the CA-BAT, the BDAT uses a one-alternative forced-choice (1-AFC) task paradigm: participants listen to each stimulus once rather than making a judgment comparing two stimuli as in the CA-BAT. This makes trials comparatively short and reduces the working memory load on participants. Finally, the BDAT allows for investigation of the impact of implied meter on beat perception in the absence of local acoustic cues of beat strength.

## Study design

The aim of Study 1 (calibration) was to construct the main BDAT paradigm and obtain data for estimating an explanatory item response theory (IRT) model. The explanatory IRT contributes to the test’s validity by assessing its assumptions and hypotheses. In addition, the explanatory IRT model provides the basis for estimating the difficulties of the test items, which are later used for estimating participant abilities in the adaptive test.

Previous research (e.g., Nguyen, 2017) suggests that beat processing abilities differ in individuals with extensive musical training versus the general public. Therefore, the secondary aim of Study 1 was to investigate whether these two groups differ in their performance on BDAT.

Study 2 aimed to explore relationships among three measures: beat perception as measured by BDAT performance, beat perception as measured by CA-BAT performance, and self-reported musical sophistication based on the Gold-MSI questionnaire. This information is important to understanding the nature of the ability that is quantified by the BDAT.

## Study 1

### Methods

#### Participants

A total of 136^1^ participants were recruited for an online experiment. Eleven participants with incomplete responses were disregarded at the data analysis stage, leaving 125 participants: 72 identified as female and 53 as male, ages ranged from 18 to 66 years (*M* = 30.06, *SD* = 8.79). Participants were recruited through social media and email invitations. The sample consisted of 40 (32%) self-declared musicians, 19 (15.2%) dancers, 64 (51.2%) individuals who did not identify as musicians or as dancers, and two (1.6%) individuals who preferred not to disclose this information. Participants were considered musicians or dancers if they were currently studying for a music or dance degree at a higher education institution, if they had graduated from such an institution, or if they had more than 10 years of experience actively engaging in music or dance activities in a professional or semiprofessional setting. All participants provided informed consent to participate in the study.

#### Materials

##### BDAT

Thirty novel musical tracks were composed and synthesized by the first author in the style of contemporary electronic dance music (EDM) using Ableton Live 10. Clips were created to be naturalistic musical stimuli without any vocals, and therefore included diverse instrumentation and sound effects, and employed a variety of tonalities. The sound was the same in both left and right channels. All musical events were aligned to a MIDI grid to ensure timing precision. All clips consisted of 6 bars of music and were composed using the time signature of 4/4 in the tempo of 125 bpm (i.e., 480 ms between beats), which resulted in clips of approximately 11.5-s duration. Common time (4/4) was chosen as a norm of contemporary dance music, and the tempo (which is in the range of EDM heard in clubs) was decided based on the results of a small pilot study that identified 125 bpm as the most natural-sounding tempo for the chosen musical style.^2^

**Fig. 1:**
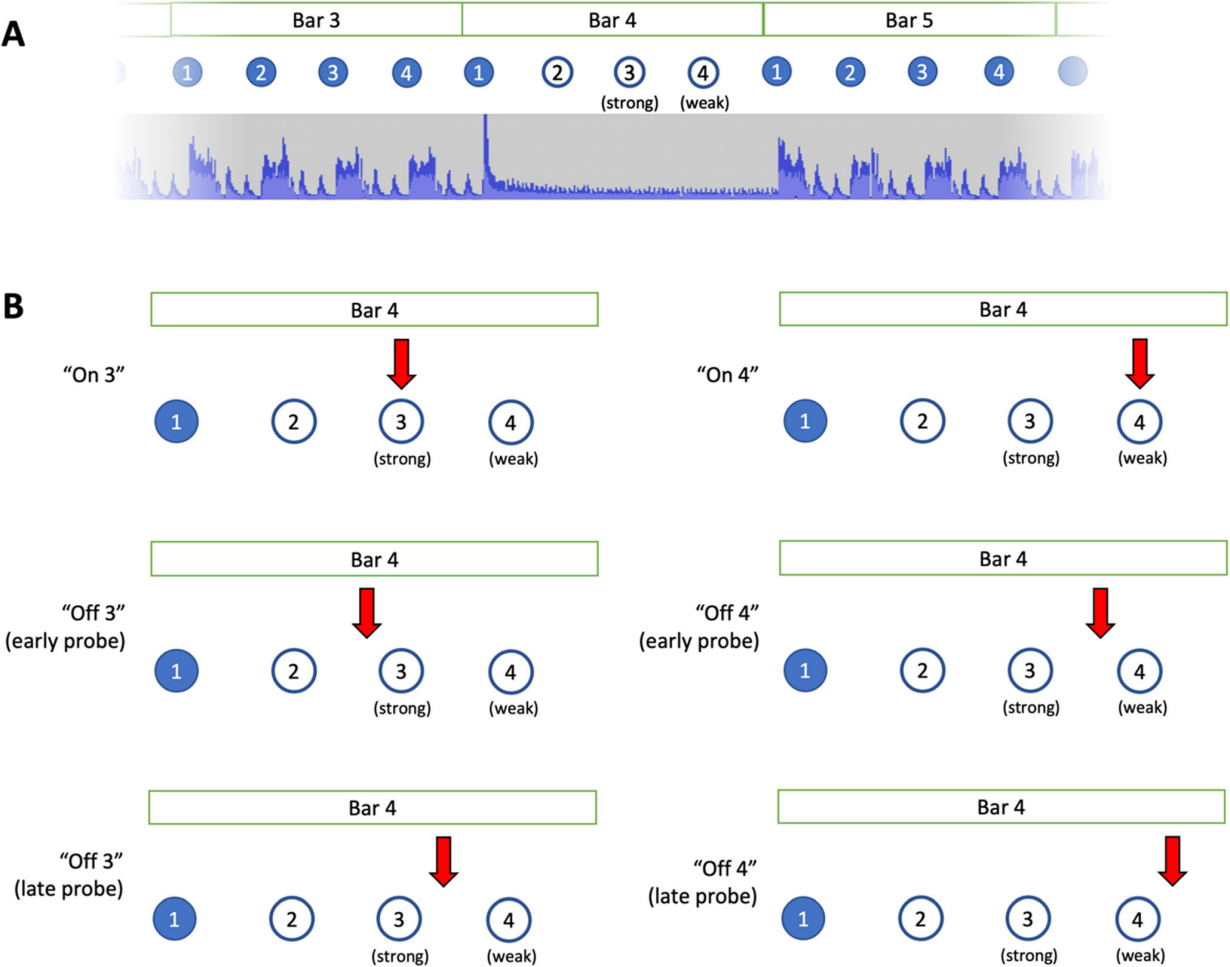
Structure of BDAT stimuli. **a** Absence of rhythmic musical cues in Bar 4, Beats 2–4. **b** (Top line): Possible locations of on-beat probes; Lines 2–3 show possible locations of off-beat probes. For visual clarity, off-beat probes are only shown in one location either early or late relative to Beats 3 or 4, but seven locations were probed in each of these cases (see text). (Colour figure online)

All clips were structured in the following way: musical material was introduced in Bars 1–3, Bar 4 contained the beat-drop (from Beats 2–4) and a probe sound during this beat-drop that was either aligned or misaligned with the underlying beat, and in Bars 5–6 the musical material returned. (The probe sound was a 16^th^-note duration woodblock pitched at F2, duration = 120 ms; Fig. 1). A beat-drop is a sudden absence of most sounds for a short period of time, most commonly used as a compositional device to create musical tension in electronic dance music. During the beat drop there were no rhythmic cues, though rhythmically ambiguous sound (e.g., a drone) was present to fill the four-beat gap and to help build expectation for the return of the rhythm.

The probe in the fourth bar was placed to either coincide with the beat of the music (ON condition), or it was shifted away from the beat (OFF condition). In the ON condition, the probe was placed on the 3^rd^ (strong) beat or the 4^th^ (weak) beat (Palmer & Krumhansl, 1990). In the OFF condition, variations were created by manipulating the following parameters: metrical strength (strong/weak; probe was manipulated away from the 3^rd^ or 4^th^ beats), probe displacement direction (early/late; the probe would be placed either before or after the beat), probe displacement (degree of displacement from the actual beat location; see Fig. 1 and supplementary sound examples).

##### Probe displacement settings

Probe displacement was measured in units of beat period and ranged from 15% to 45% of the beat period in 7 steps. As noted in Harrison and Müllensiefen (2018), the relationship between probe displacement (in % of units of a beat) and perceptual difficulty is not linear (see Harrison & Müllensiefen, 2018, for details), therefore probe displacement points were established by finding equal distances on the perceptual accuracy scale that accounts for the relationship between physical probe displacement and perceived difficulty, and is expressed by the following formula:

1 $$ \mathrm{Perceptual}\ \mathrm{accuracy}=\cos \left({\mathrm{pi}}^{\ast}\mathrm{displacement}\right)\hat{\mkern6mu} 4. $$

Expressed as a percentage, these calculations resulted in the following displacement points: 15%, 18%, 20%, 23%, 26%, 31%, 45%. These levels were chosen as they were above the displacement hearing threshold (Harrison & Müllensiefen, 2018) and below a displacement of 50% (since an event occurring exactly halfway between beats, a frequent location for rhythmic events, might be mistaken for “on the beat”). Also, a 50% displacement following the 3^rd^ beat coincides with a 50% displacement preceding the 4^th^ beat, and such conceptual overlap would interfere with later analyses.

Variations of the experimental stimuli were created using automated item generation coded in Python. 2 ON and 28 OFF variations were produced for each of the 30 tracks, which yielded a total of 900 items (audio clips).

As noted earlier, a 1-AFC paradigm was employed for the test: participants listened to 30 clips of music and answered whether the probe was on or off the beat. Every correct response scored 1 point, and every incorrect response scored 0. Participants heard each of the 30 tracks once. Each track was presented in either ON or OFF condition with a 50% chance. The perturbation for the OFF tracks was chosen randomly from the 28 options: seven options with the probe coming before Beat 3 (early/strong beat), seven options with the probe coming after Beat 3 (late/strong beat), seven options with the probe coming before Beat 4 (early/weak beat), and seven options with the probe coming after Beat 4 (late/weak beat).

The BDAT can be downloaded and installed (https://github.com/klausfrieler/BDT/). All BDAT materials can be accessed online (https://osf.io/jpc29/).

##### Gold-MSI

To evaluate the level of musical sophistication of the participants, the Goldsmiths Musical Sophistication Index was used (Gold-MSI; Müllensiefen et al., 2014). The Gold-MSI assesses a broad range of self-reported musical skills and behaviours on five dimensions: active engagement, perceptual abilities, musical training, singing abilities, and emotions. Study 1 only made use of two subscales: Musical Training (Gold-MSI_MT), composed of seven items assessing the extent of musical training and practice, and Perceptual Abilities (Gold-MSI_PA) composed of nine items that represent the self-assessment of cognitive and perceptual musical abilities. These scales can be downloaded (http://gold-msi.org).

#### Procedure

The experiment was conducted online using the Qualtrics survey platform. Participants provided their informed consent and familiarized themselves with the task by listening to a sample track presented first in the ON condition and then in the OFF condition. They were asked to use headphones and were asked not to tap or otherwise move to the beat of the music, as movement to the music has been demonstrated to affect beat perception (Manning & Schutz, 2013; Morillon et al., 2014). The BDAT was followed by the Gold-MSI questionnaire. Participants were also asked to rate test difficulty on the 7-point Likert scale, with 1 being *easy* and 7 being *extremely difficult*.

### Results

#### Preliminary analyses

A total of 104 individuals completed Study 1 in full, and 21 more responded to 29 out of 30 stimuli, so their data were included in the analysis. There were no missing data on demographics, Gold-MSI questionnaire, or test difficulty rating. Difficulty ratings varied from 1 to 7 (*M* = 4.28, *SD* = 1.59), where 1 meant *very easy* and 7 meant *extremely difficult*. Difficulty ratings correlated with the level of musical training, with musically trained participants rating the test as easier than musically untrained participants (*r* = −.406, *p* < .001). Raw test scores ranged from 7 to 30 correct out of 30 trials (*M* = 18.74, *SD* = 3.96). Across participants, the correlation between difficulty ratings and raw test scores was significant (*r* = −.216, *p* = .016), indicating that higher perceived difficulty ratings were associated with poorer performance on the test.

Participant response reliability was checked by investigating whether any of the participants provided all/nearly all ‘ON’ or all/nearly all ‘OFF’ answers; no such participant was identified, suggesting that the test was taken seriously.

Gold MSI Music Training scores varied from 7 to 46 (*M* = 27.41, *SD* = 12.85; scoring range 7 to 49), Gold MSI Perceptual Abilities scores varied from 22 to 62 (*M* = 47.60, *SD* = 8.51; scoring range 9 to 63). These values resemble published norms drawn from a sample of nearly 150,000 participants from the general population (*M* = 26.52, *SD* = 11.44 for Musical Training and *M* = 50.20, *SD* = 7.86 for Perceptual Abilities; Müllensiefen et al., 2014). A highly significant correlation was found between BDAT raw scores and Gold-MSI self-report questionnaire scores on subscales Musical training and Perceptual Abilities (*r* = .324, *p* < .001; *r* = .297, *p* < .001 respectively).

#### Responses to individual tracks

The average response accuracy for each of the 30 tracks was computed by counting the number of correct responses to that track across all probe positions tested, divided by the total number of responses to that track. All accuracies were above the chance level of 0.5 (range = .53–.744, *M* = .63, *SD* = .48). However, track averages differed when calculated for ON and OFF conditions separately. In the ON condition overall accuracy was higher (*M* = 0.67, *SD* = .47), and only one track (Track 3) had below chance performance (*M* = .42, *SD* = .50). In the OFF condition overall accuracy was lower than in the ON condition (*M* = .58, *SD* = .49), with three tracks showing below-chance accuracy levels (Track 12, *M* = 0.48, *SD* = 0.50; Track 27, *M* = 0.48, *SD* = 0.50; Track 30, *M* = 0.49, *SD* = 0.50; for accuracy data on individual tracks, see figures in Supplementary Information). Because below chance averages can be indicative of a stimulus bias, Track 3 was excluded from further analysis of the ON condition, and Tracks 12, 27, and 30 were excluded from further analyses of the OFF condition.

#### Effect of participant group: Differences between musically trained and general public.

**Fig. 2:**
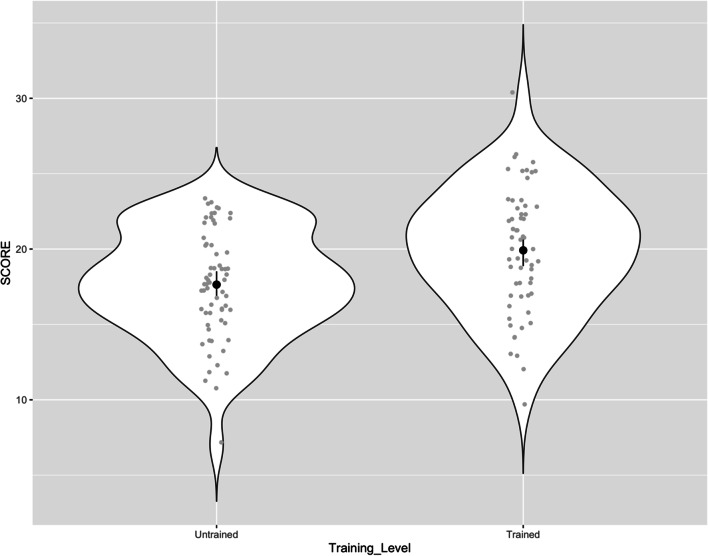
Distribution of scores for two groups—general population (untrained) and musically trained people. Maximum score = 30. *Note*. The raw data point appearing slightly above 30 is due to the jitter that is applied to make the raw data more visible. The data is jittered along the *x*-axis and along the *y*-axis, and the latter lifts this observation with a true score of 30 slightly above the 30-points line

Study 1 had a sample of 40 musicians, 19 dancers, and 64 individuals from the general public. Two people preferred not to disclose this information and were therefore removed from the analysis of the effect of group. A nonsignificant Levene’s test showed no violation of the equality of variances assumption, *F*(2, 120) = .326, *p* = .723. A one-way analysis of variance (ANOVA) detected a significant effect of group, *F*(2, 120) = 6.04, *p* = .003; however, analysis of contrasts showed no significant difference between dancers and musicians, *t*(120) = −1.12, *p* = .265, who were therefore pooled into a ‘musically trained’ group (*N* = 59) and compared with the general public (*N* = 64). Musically trained people differed from the general public, *t*(120) = 2.88, *p* = .005, with the former scoring higher (*M* = 19.92, *SD* = 4.09 vs. *M* = 17.64, *SD* = 3.59; Fig. 2, cf. Supplementary Information Tables S1–S4).

#### Explanatory item response modeling

For modeling item difficulty at the level of the individual trial, we employed the approach outlined in Harrison and Müllensiefen (2018) and Larrouy-Maestri, Harrison & Müllensiefen (2019), based on the explanatory item response modeling framework laid out in De Boeck and Wilson (2004). The item response model took the form of a generalized mixed-effect model with a logit link function (also known as mixed-effect logistic regression model) and modified asymptotes (Harrison & Müllensiefen, 2018). This model reproduces a four-parameter logistic IRT model in which the guessing, discrimination, and inattention parameters are constrained not to vary within the item bank (Magis & Raîche, 2012). In addition to calibrating the difficulty of the individual items, the explanatory item response model also allows one to quantify the relationship between item difficulty and the structural item features (probe displacement, probe direction, strength of target beat), which is useful for investigating the test’s assumptions and contributing to its construct validity. Generalized mixed-effects models were computed separately for ON and OFF conditions.

##### ON condition

The model for the items in the ON condition included the binary response accuracy (0 or 1) as dependent variable, metrical strength as the only fixed effect (binary, with Target Beat 4 coded as *weak* and Target Beat 3 coded as *strong*), and participant ID as well as track as a random effect. The model was fit using the functions glmer() from the R package lme4 and logit.2asym() from the R package psyphy. A summary of the model is given in Table 1, which shows a significant effect for strong beats, with the performance being better on strong beats (76.5% correct) than on weak beats (57.5% correct). The model for the ON condition achieved a prediction accuracy of 71.2%.

**Table 1:** Generalized linear mixed-effects model: Condition ON

Effect	Type	*SD*	*B*	*SE*(*B*)	*z*	*p*
ID	Random	1.19				
Track	Random	0.41				
Intercept	Fixed		−0.76	0.21	−3.65	<.001
Beat strength	Fixed		1.77	0.24	7.53	<.001

The estimated value for the lower asymptote (guessing) of the mixed-effect logistic regression model is .4, and the value for the upper asymptote (inattention) of the model is .98

##### OFF condition

The model for the items in the off condition also used response accuracy as a dependent variable, with metrical strength, probe direction (binary, with categories before and after target beat), probe displacement (numerical variable with seven levels, ranging from 15% to 45% displacement), and interaction between metrical strength and direction as fixed effects, and with track and participants as random effects. The model summary in Table 2 shows that all fixed effects except metrical strength make a significant contribution. However, because the interaction term Probe Direction × Metrical Strength is significant, we also kept the main effect of metrical strength in the model. The model for the OFF condition items achieved a prediction accuracy of 71.3%.

**Table 2:** Generalized linear mixed-effects model: Condition OFF

Effect	Type	*SD*	*B*	*SE*(*B*)	*z*	*p*
ID	Random	1.30				
Track	Random	0.05				
Intercept	Fixed		2.23	0.38	5.88	<.001
Displacement	Fixed		0.41	0.07	−5.61	<.001
Direction	Fixed		−1.91	0.36	−5.32	<.001
Beat strength	Fixed		0.16	0.33	0.50	.619
Direction × Beat Strength	Fixed		−1.48	0.56	−2.63	.009

The estimated value for the lower asymptote (guessing) is .4, and the value for the upper asymptote (inattention) is .96

**Fig. 3:**
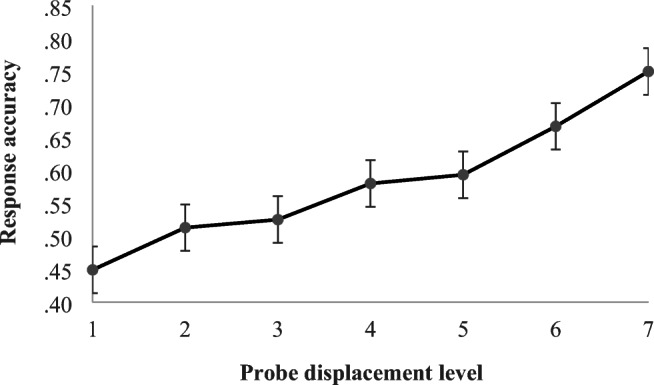
Mean response accuracy increases with probe displacement

The plot of the mean response accuracy across the seven levels of probe displacement in Fig. 3 shows an approximately linear trend, which confirms that a transform for the linear predictor displacement is not necessary.

### Discussion

Study 1 aimed to (1) determine if participants could do the BDAT without experiencing it as overly difficult, (2) identify any problematic test items, (3) investigate whether the BDAT is sensitive to the degree of musical training, (4) obtain data for estimating IRT parameters for the subsequent construction of an adaptive version of the BDAT, and (5) see if metrical strength influenced accuracy in judging alignment of a probe sound with a beat, which had not been examined before with real music.

We found that participants could do the BDAT and rated it as moderately difficult (mean of 4.3 on a scale of 1–7). As expected, participants with extensive musical training (dancers and musicians) performed significantly better than the general public, with mean raw scores about 13% higher than the general public (Fig. 1). A significant association between participant test score and Gold-MSI scores indicated that participants with a higher level of musical training and higher self-reported perceptual abilities were better at this beat perception task. The correlation levels were comparable, though slightly lower, than that found between Gold-MSI Musical Training and CA-BAT performance in previous work, *r*(195) = .454, *p* < .001 (Harrison & Müllensiefen, 2018). Such a moderately-sized correlation was expected because beat perception abilities are assumed to depend moderately on musical training (i.e., less strongly associated with musical training than, for example, melodic discrimination ability; Harrison & Müllensiefen, 2018). This could be interpreted as an argument towards beat perception abilities being influenced by genetic predispositions (cf. Niarchou et al., 2022.

Both explanatory item response models showed acceptable predictive accuracy. The model for items in the ON condition showed that metrical strength was a significant factor determining response accuracy, with better performance when the probe was placed on the strong beat. This result is expected in light of the work of Palmer and Krumhansl (1990), who found that probe events placed on strong beats were rated as fitting an ongoing rhythmic pattern better than probe events on weak beats.

For the OFF condition, response accuracy was significantly higher with larger probe displacement, as expected, with a roughly proportional association between response accuracy and degree of displacement. Probe displacement direction was also a significant determinant of response accuracy: Accuracy was higher when the probe was placed ahead of rather than after the target beat location. This is the opposite pattern of results to that found in an earlier Beat Alignment Test study with 15 professional musicians (Van Der Steen et al., 2014) but accords with musical intuition since certain musical styles favor rhythmic delays rather than musical events ahead of implied beat locations (e.g., delayed snare in swing; Butterfield, 2010). It also agrees with two reported timing psychophysics results. First, the “filled duration” illusion causes listeners to perceive an empty interval as shorter than a subdivided interval (Repp, 2008; Repp & Bruttomesso, 2009); this would lead listeners to perceive slightly late events following empty intervals as on time, as we observed. Second, listeners experience “perceptual acceleration” during isochronous sequences, showing a bias toward reporting the last interval as slightly short (Li et al., 2016); this, too, would lead listeners to perceive late events as on time.

Metrical strength of the beat was a non-significant factor on its own in the model for OFF items, but its interaction with direction was significant: participants performed better when the probe came late on the weak beat than when it came late on the strong beat. Again, this could be interpreted as listeners being more tolerant of slightly delayed musical events especially when they come after strong beats, as this is a musical feature in certain styles where such delays can create the impression of a ‘laid back’ feel. Another possible explanation is that probes falling late on the weak beat were the closest to the point at which the rhythm reenters and could be more easily recognized as off the beat by comparing their timing to the timing of rhythm reentry.

## Study 2

Study 2 aimed to conduct a correlative investigation of the relationships among three scores: the BDAT, the CA-BAT, and self-reported musical sophistication as quantified by the Gold-MSI questionnaire (Müllensiefen et al., 2014). Our goal was to better understand the aspect(s) of beat perception quantified by BDAT performance.

### Methods

#### Participants

A total of 103 participants were recruited for this online study. Two participants reported hearing impairments and were therefore excluded from the analysis. In the remaining sample of 101 participants, 52 identified as female, 47 as male, and two chose not to disclose this information. Participants were ages 21 to 63 years (*M* = 29.91, *SD* = 7.21).

Participants were recruited through social media and email invitations. All participants provided informed consent to participate in the study.

#### Materials

A three-part battery was constructed for Study 2, consisting of an adaptive version of BDAT, the Computerised Adaptive Beat Alignment Test (CA-BAT; Harrison & Müllensiefen, 2018), and the full version of the Gold-MSI questionnaire (Müllensiefen et al., 2014), including all five subscales as detailed below.

##### BDAT

Study 2 used the same musical clips as Study 1, with the stimulus set expanded to include tempo variation as an additional random factor. Slight variation in tempo ensured that participants had to identify the tempo every time they listened to a new clip. Thus, their perceived tempo as well as the phase of the beat were based entirely on the clip they were hearing and not on a developed expectation that the tempo would always be fixed.

There were five tempo variations of each clip, which produced 4,500 files. Tempo varied in even steps ±5% from the original 125 bpm, producing 119 bpm, 122 bpm, 125 bpm, 128 bpm, 132 bpm; 5% tempo steps provided five perceptually distinct tempi but did not distort the musical material.

Following track-reliability analysis of Study 1, Study 2 eliminated the usage of Track 3 in ON condition and Tracks 12, 27, and 30 in OFF condition.

For Study 2, an adaptive version of BDAT was created on the basis of the explanatory IRT models for ON and OFF conditions computed in Study 1. In this adaptive version, on each new trial the difficulty of the item presented was matched to participant performance that was estimated dynamically after each trial. This was based on the item difficulty parameters as computed for all items in Study 1 (see Harrison & Müllensiefen, 2018, for details of item difficulty computations). In addition, the standard deviations of the participant random intercepts were extracted as constant discrimination parameters for ON and OFF items. Similarly, the parameters for lower and upper asymptotes were used as constants across all items. In IRT models, person abilities, as well as item difficulties, are defined on the same metric, a *z*-score scale typically ranging from −4 to 4. This means that, for example, a participant with an ability score of 1 is one standard deviation above the population mean. The first item of the adaptive test was always chosen to have the difficulty level 0, matching the average ability of the participant sample from Study 1.

##### CA-BAT

The purpose of this test is to evaluate the listener’s beat perception ability using an adaptive version of the beat alignment test (BAT) which tailors the difficulty level of the test to each participant by adapting to their previous responses (Harrison & Müllensiefen, 2018). This maximizes the amount of information that each successive item gives about participant’s actual ability, which in turn ensures shorter testing time and thus improves test’s efficiency. Similar to the BDAT, the CA-BAT uses naturalistic music stimuli. In contrast to the BDAT, the stimuli were not created specifically for the test (see Harrison & Müllensiefen, 2018, for details), meaning that minor temporal fluctuations are probable within items. However, this is mitigated by using a 2-AFC paradigm. For each trial, the participant is presented with two versions of a musical track, both overlaid with a metronomic probe track. The ON version of the track has the probe in time with the musical beat locations while the OFF version has the probe track displaced from the musical beat locations. The participant’s task is to identify the ON track. Person ability score for CA-BAT is computed following the same principles as for the adaptive BDAT, described above.

##### Gold-MSI

Study 2 employed the full version of the questionnaire, which assessed all five subscales (Active Engagement, Perceptual Abilities, Musical Training, Emotion, Singing Abilities) as well as the General Musical Sophistication (GMS) scale that draws on items from all five subscales. Altogether, the Gold-MSI self-report questionnaire was composed of 41 questions.

#### Procedure

Study 2 was conducted online using an interface based on the open-source psychTestR package (Harrison, 2020). All participants provided their consent for taking part in the study. They were asked to wear headphones for the entire duration of the test and adjust their volume to a comfortable level. As in Study 1, participants were asked not to tap or otherwise move to the beat of the music.

The battery started with the collection of demographic information and was followed by the BDAT (25 items), Gold-MSI questionnaire (41 questions), and CA-BAT (25 items).^3^ Prior to each test participants were presented with a training phase which included instructions, two example stimuli (one for Condition ON and one for Condition OFF), and two practice items. The reported testing time was 25–30 minutes. Completion of the test led to the display of task performance as pseudo-IQ scores in a numerical as well as graphical format (bell curve with the mean of 100 and the standard deviation of 15), alongside the General Musical Sophistication (GMS) score.

### Results

**Fig. 4:**
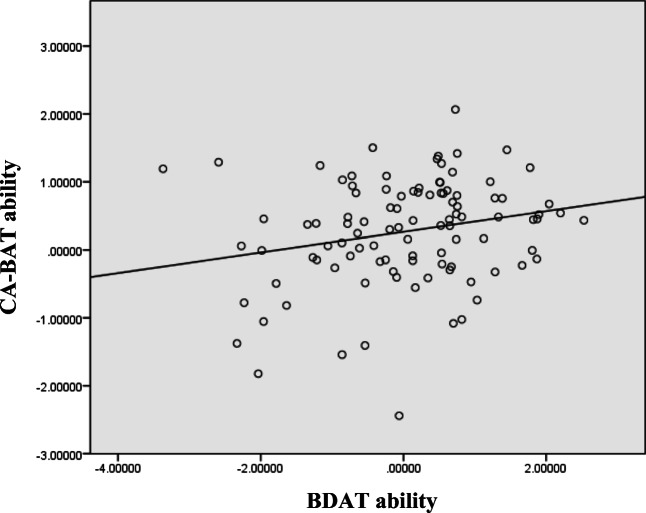
Correlation between BDAT and CA-BAT ability

BDAT ability scores varied from −3.37 to 2.53 (*M* = 0.04, *SD* = 1.17), CA-BAT ability scores varied from −2.44 to 2.07 (*M* = 0.27, *SD* = 0.78), and GMS scores varied from 1.45 to 6.45 (*M* = 4.23, *SD* = 1.18). For comparison, published GMS norms vary from 1 to 7, *M* = 4.53, *SD* = 1.15 (Müllensiefen et al., 2013). Significant correlations were found between BDAT and CA-BAT scores (*r* = .23, *p* = .023; Fig. 4) as well as between BDAT and GMS scores (*r* = .55, *p* < .001). Indeed, BDAT scores were highly significantly correlated with all the dimensions of the Gold-MSI questionnaire, the strongest correlations being with Musical Training and overall GMS score.

CA-BAT scores were also significantly correlated with all of the Gold-MSI dimensions except Singing Abilities, though in most cases less strongly than BDAT scores. The highest correlation was found with the dimension Active Engagement (Table 3).

**Table 3:** BDAT and CA-BAT correlations with dimensions of Gold-MSI

	Active engagement	Musical training	Emotions	Singing abilities	Perceptual abilities	GMS
BDAT	.313	.513	.348	.499	.456	.545
*p*	<.001	<.001	<.001	<.001	<.001	<.001
CA-BAT	.325	.200	.191	.136	.260	.266
*p*	<.001	.022	.028	.088	.004	.004

*GMS* general musical sophistication, which draws on items form all subscales in Columns 1–5. The table displays Pearson correlations with the one-tailed significance levels

### Discussion

The purpose of Study 2 was to characterize relationships among BDAT performance, CA-BAT performance, and Gold-MSI questionnaire scores. Inspecting the correlations between Gold-MSI dimensions and BDAT scores shows that the overall GMS score, and musical training in particular, partially predicted performance on the BDAT’s covert beat continuation and comparison task. Regression analysis showed that the Gold-MSI subscales collectively accounted for around one third of the variation in BDAT scores (*R*^2^ = .34), indicating that while beat perception ability as indexed by the BDAT was influenced by (or possibly influenced) musical training and sophistication, it also seemed to reflect skill or ability that was not solely based on training or engagement. However, the modest correlation between BDAT and CA-BAT scores suggested that this skill or ability was not identical to that measured by the CA-BAT, which was less correlated with the GMS score and most of its subfactors, including musical training.

Overall, these results indicate that the aspect of beat perception tested by the BDAT is more closely related to general music skills than the aspect tested by the CA-BAT. To make such a conclusion with confidence, it would be important to estimate the degree to which variability in each test reflects measurement error vs. a consistent attribute of the participant, and to observe the changes of both scores with musical training interventions.

## General discussion

This study developed and explored a new test of musical beat perception which does not rely on synchronized movement to the beat. The primary innovation of the BDAT is that it tests musical beat perception in the absence of any sensory cues to the beat. In the BDAT, the listener hears a few bars of beat-based music and then judges if a single probe event is on or off the beat during a “beat drop” when all rhythmic cues to the beat have been removed. Thus, the BDAT requires the listener to continue a beat percept formed while hearing rhythmic music through a beat-drop bar until the music resumes. The BDAT was created to provide a focused test of the capacity to covertly continue a beat, which cannot be directly investigated by tests like the BAT, which allow for the use of local acoustic cues in judging timing. Indeed, the results of our study of the BDAT suggest that the BDAT is testing aspects of beat perception that are partly independent from those tested by the BAT, as we discuss below.

Among other tests of rhythm perception, the BDAT has several advantages. It uses a variety of realistic musical materials composed in the style of electronic dance music (created specifically for the test), is quick to administer, and has beats at unambiguous locations (since the music was composed using a MIDI time grid). Furthermore, it can be used to study beat perception as a function of metrical position (strong vs. weak beats), direction of probe misalignment (early vs. late), and degree of probe displacement from beats.

The current study examined performance on the BDAT in two experiments. The first experiment showed that the BDAT was not rated as highly difficult by participants, and that individuals with a high degree of musical training scored significantly better on the test. However, overall performance was generally low, on average around 60%–70% correct. This is not surprising given that each trial of the BDAT has a single probe sound, unlike the BAT, in which there is an entire metronomic train of probe sounds. Furthermore, when the probe sound was misaligned, it was often very close to a beat location, making the misalignment difficult to detect (Fig. 2). Restricting off-beat probes to larger displacements should result in higher overall BDAT performance scores in future work.

A novel finding of experiment 1 is that accuracy in judging when a probe event is on the beat differed substantially depending on whether the probe was on a strong versus weak metrical position (Beat 3 vs. 4) in the beat drop bar, with accuracy about 20% higher on the strong beat (76.5% vs. 57.5% correct). Interestingly, the music for our study was not composed in a way to acoustically emphasize strong versus weak beat positions, suggesting a potentially significant role for top-down metrical expectations in shaping our results. Our finding aligns with research by Palmer and Krumhansl (1990), who had listeners listen to metronomic sequences of equal-loudness events and imagine different meters, and who found that probe events placed on metrically strong beats were rated as fitting better with the rhythm than events on weak beats. However, the differences in their probe ratings were subtle compared with the large effects seen in the current study. Further work is needed to determine if the large accuracy difference we see on strong versus weak beats is due to metrical expectations (cf. Iversen et al., 2009), or simply reflects the fact that our weak beat position was later in the beat drop bar than the strong beat position. Due to this design, any internally maintained pulse might be diminished and/or less precise at the time of the weak versus strong beat simply by virtue of the greater time elapsed since the cessation of rhythmic cues to beat structure (Cannon, 2021). In future work it would also be interesting to determine if the metrical effect we see only emerges after a certain age, reflecting the development of metrical knowledge of the culture’s prevailing musical patterns (Nave-Blodgett et al., 2021).

The second experiment in our study used data on item difficulty taken from the first experiment to eliminate a few overly difficult stimuli and to create an adaptive version of the BDAT, in which item difficulty increased as the experiment progressed. Experiment 2 also introduced slight intertrial tempo variation between stimuli to force listeners to infer the tempo of the beat on each trial rather than forming an experiment-wide tempo prior that could influence task performance. All participants in this experiment were also tested on a version of the CA-BAT (the computerized-adaptive BAT) and were given the full Gold-MSI questionnaire to measure musical sophistication, including five subscales (Active Engagement, Perceptual Abilities, Musical Training, Emotion, Singing Abilities). This experiment found that performance on the BDAT and CA-BAT showed only a modest correlation, and that the extent to which the two tests correlated with subscales of the Gold MSI and with general musical sophistication (GMS) differed substantially. For example, self-reported singing abilities correlated with the BDAT performance but not CA-BAT performance. Indeed, correlations between BDAT performance and *all* subscales of the Gold-MSI (and with GMS) were substantially higher than were correlations with CA-BAT performance (Table 3), suggesting that performance on the BDAT may be tied to a wide range of musical abilities.

One musical ability likely to be relevant to the BDAT is musical imagery during the beat-drop bar. Neuroimaging research has shown that detailed imagery for musical patterns involves a complex network of brain regions aside from regions involved in beat processing (Cannon & Patel, 2021; Regev et al., 2021). Furthermore, musical imagery abilities and pitch imitation abilities appear to be related (Greenspon et al., 2017), which could help explain why self-related singing abilities correlate with BDAT performance if listeners engage in musical imagery during the beat drop. Another relevant musical ability might be motoric continuation of a beat: Although participants were asked not to move, subtle movements and/or motor imagery may have played roles in performance. In future work, it would be interesting to determine if BDAT performance is associated with individual differences in auditory imagery abilities (e.g., as measured by the Bucknell Auditory Imagery Scale; Halpern, 2015), and in the continuation phase of a synchronization-continuation task.

Another factor that could help explain the relatively low correlation between BDAT and CA-BAT performance is the fact that the latter involves a memory component since two musical clips must be compared with determine which has on-beat beeps. The BDAT, like the original BAT, only requires listening to each clip once. Another difference could be the additional auditory processing required in the CA-BAT in order to compare the musical rhythm to a concurrently presented series of beeps. More generally, it appears that the BDAT provides a distinctive test of beat perception, engaging some different cognitive processes than the CA-BAT. Some of these processes may be specific to the continuation of a beat (as opposed to the initial recognition of the beat).

The BDAT offers a range of possible applications in the study of human auditory rhythmic processing. Researchers using the BDAT can decide whether to use BDAT stimuli that are uniform in tempi (as in Experiment 1) or slightly different in tempi from trial to trial (as in Experiment 2), depending on their goals. Furthermore, the BDAT stimuli, which are freely available, may prove useful in a range of studies of beat perception, including neural studies aimed at studying oscillatory neural dynamics during beat perception. (To facilitate such work, the online data archive for this paper includes versions of the BDAT stimuli without probe sounds). Due to the novel beat-drop design of the BDAT, any beat-related neural oscillations during the beat-drop bar cannot be due to stimulus-driven brain activity, and this could help test existing models of the causes of beat-related neural oscillations in the brain (Breska & Deouell, 2017; Doelling & Assaneo, 2021; Tal et al., 2017). By placing beat drops of predictable duration at musically appropriate times and filling the gap with appropriate nonrhythmic musical content, these stimuli induce a strong expectation of the return of the rhythm. BDAT stimuli may thus have some advantages for studying perceptual and neural oscillations involved in rhythm perception compared with stimuli used in the past, which examine activity after the sudden end of a rhythmic stimulus (Hickok et al., 2015; Stupacher et al., 2013; van Bree et al., 2021). The BDAT stimuli may also prove useful in future neural studies of musical imagery, if such imagery is indeed one cognitive tool that participants use to do the task. Finally, like one current use of the BAT, the BDAT may prove to be a useful tool for studies with patients with movement disorders or neurodegenerative diseases. Based on our findings we feel that the BDAT is a viable and novel instrument for exploring beat perception, and merits further study and development.

## Supplementary information

ESM 1 (DOCX 127 kb)

ESM 2 Track 9. Probe discrepancy by 45%, placed before the 3rd beat of the beat-drop bar. (MP3 449 kb)

ESM 3 Track 5. Probe on the 3rd beat of the beat-drop bar. (MP3 449 kb)

ESM 4 Track 27. Probe discrepancy by 45%, placed after the 3rd beat of the beat-drop bar. (MP3 449 kb)

ESM 5 Track 24. Probe discrepancy by 45%, placed after the 4rd beat of the beat-drop bar. (MP3 449 kb)

ESM 6 Track 19. Probe discrepancy by 45%, placed before the 4rd beat of the beat-drop bar. (MP3 449 kb)

ESM 7 Track 12. Probe on the 4th beat of the beat-drop bar. (MP3 449 kb)

## Footnotes

1 Only those participants who started the listening session are reported. This number does not include additional 29 participants who dropped out before starting the listening session.

2 In the pilot study, seven individuals were presented with nine clips: three tracks in three different tempi (namely, 120 bpm, 125 bpm, and 130 bpm).

3 25 BDAT items were used in this experiment to match the number of tracks on the CA-BAT. Of the original 30 BDAT source stimuli, four were eliminated due to below-chance performance in Experiment 1, and 25 of the remaining 26 were randomly chosen for each participant in Experiment 2.
