# Supplementary material for: Testing beat perception without sensory cues to the beat: the Beat-Drop Alignment Test (BDAT)
Source: Atten Percept Psychophys. 2022 Oct 19;84(8):2702–14. doi: 10.3758/s13414-022-02592-2 (PMC9630205; doi:10.3758/s13414-022-02592-2)
Supplement: Supplementary file 1 — (DOCX 127 kb) [file 13414_2022_2592_MOESM1_ESM.docx]

Supplementary Information

Study 1: Response accuracy to individual tracks

**Figure S1**

*Mean response accuracy to individual tracks: both conditions (max = 1.0)*


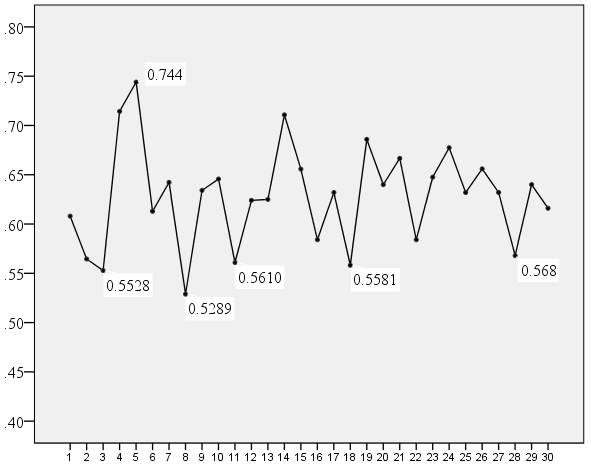


**Track**

**Mean response accuracy**

A

**Figure S2**

*Mean response accuracy to individual tracks: condition ON (max = 1.0)*


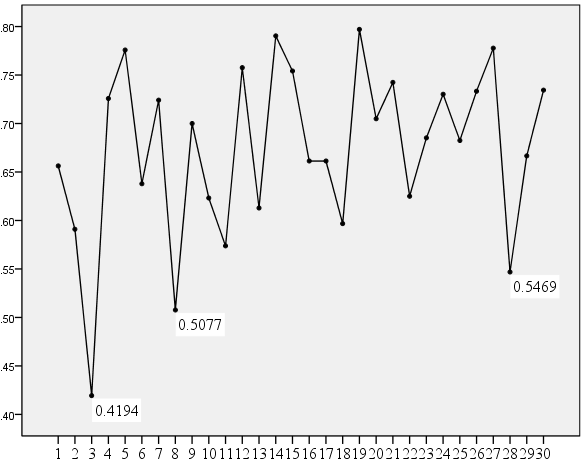


**Track**

**Mean response accuracy**

**Figure S3**

*Mean response accuracy to individual tracks: condition OFF (max = 1.0)*


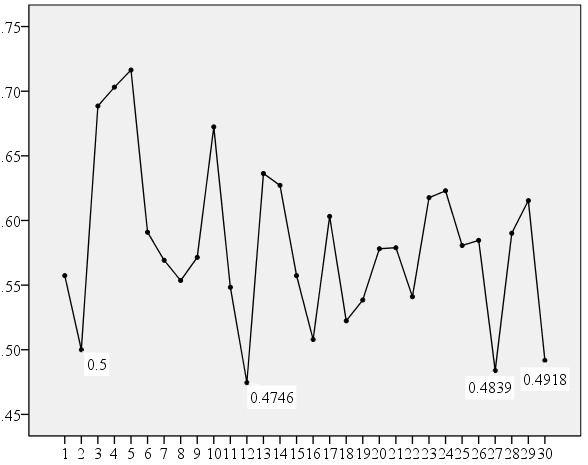


**Mean response accuracy**

**Track**

**Mean response accuracy**

|  | | | | | | | | |
| --- | --- | --- | --- | --- | --- | --- | --- | --- |
| Table S1  *Effect of group on total test score. Descriptives* | | | | | | | | |
|  | N | M | SD | SE | 95% CI for Mean | | Min | Max |
|  |  |  |  |  | Lower Bound | Upper Bound |  |  |
| General public | 64 | 17.64 | 3.587 | .448 | 16.74 | 18.54 | 7 | 23 |
| Dancers | 19 | 19.11 | 4.175 | .958 | 17.09 | 21.12 | 12 | 30 |
| Musicians | 40 | 20.30 | 4.040 | .639 | 19.01 | 21.59 | 10 | 26 |
| Total | 123 | 18.73 | 3.986 | .359 | 18.02 | 19.44 | 7 | 30 |

Study 1: Effect of group

.

|  |  |  |  |
| --- | --- | --- | --- |
| Table S2  *Test of Homogeneity of Variances in total group scores* | | | |
| Levene's Statistic | df1 | df2 | Sig. (2-tailed) |
| .326 | 2 | 120 | .723 |

| Table S3  *Analysis of group score variance* | | | | | |
| --- | --- | --- | --- | --- | --- |
|  | Sum of Squares | df | Mean Square | F | Sig. (2-tailed) |
| Between Groups | 177.222 | 2 | 88.611 | 6.039 | .003 |
| Within Groups | 1760.924 | 120 | 14.674 |  |  |
| Total | 1938.146 | 122 |  |  |  |

Table S4

*Contrasts between groups*

| Contrast | Value of Contrast | SE | t | df | Sig. (2-tailed) |
| --- | --- | --- | --- | --- | --- |
| Dancers vs Musicians | -1.19 | 1.067 | -1.119 | 120 | .265 |
| General public vs Musically-trained | 2.06 | .717 | 2.876 | 120 | .005 |
